# Chitosan/Solid-Lipid Nanoparticles Hybrid Gels for Vaginal Delivery of Estradiol for Management of Vaginal Menopausal Symptoms

**DOI:** 10.3390/ph16091284

**Published:** 2023-09-11

**Authors:** Heba A. Abou-Taleb, Zeinab Fathalla, Demiana M. Naguib, Adel Al Fatease, Hamdy Abdelkader

**Affiliations:** 1 Department of Pharmaceutics and Industrial Pharmacy, Faculty of Pharmacy, Merit University (MUE), Sohag 82755, Egypt; heba.ahmed@merit.edu.eg; 2 Department of Pharmaceutics, Faculty of Pharmacy, Minia University, Minia 61519, Egypt; zeinab_minia_eg@yahoo.com; 3 Department of Pharmaceutics, Faculty of Pharmacy, Nahda University (NUB), Beni-Suef 62521, Egypt; demiana.monier@nub.edu.eg; 4 Department of Pharmaceutics, College of Pharmacy, King Khalid University, Abha 62223, Saudi Arabia; afatease@kku.edu.sa

**Keywords:** estradiol, permeability, irritation, vaginal deliver, menopause

## Abstract

Hormonal replacement therapy is the mainstay treatment to improve quality of life and reduce mortality. With the increasing number of young women with early menopause, women now live longer (increased life expectancy). However, poor patient compliance with oral estrogen therapy has emerged. Intravaginal estrogen therapy can provide significant benefits with minimal risk for postmenopausal women with symptoms of the lower urinary tract and vaginal area but who do not want to take oral estrogen. In this study, estradiol-loaded solid lipid nanoparticles (SLPs) were prepared from compritol ATO 888 and precirol ATO 5, and two different stabilizers (Pluronic F127 and Tween 80) were studied. Selected SLPs (F3 and F6) were coated with different concentrations of the mucoadhesive and sustained-release polymer chitosan. Furthermore, gelation time, viscosity, mucoadhesion, ex vivo permeation, and in vitro irritation for vaginal irritation were studied. Particle sizes ranged between 450–850 nm, and EE% recorded 50–83% for the six SLPs depending on the type and amount of lipids used. Cumulative % drug release was significantly enhanced and was recorded at 51% to 83%, compared to that (less than 20%) for the control suspension of estradiol. Furthermore, extensive thermal gelation and mucoadhesion were recorded for chitosan-coated SLPs. Up to 2.2-fold increases in the permeation parameters for SLPs gels compared to the control suspension gel were recorded, revealing a slight to moderate irritation on Hela cell lines. These findings demonstrated chitosan-coated estradiol SLPs as novel and promising vaginal mucoadhesive hybrid nanogels.

## 1. Introduction

Menopause describes the end of the menstrual cycle after 12 months. It is primarily due to decreased ovarian estrogen production and is associated with amenorrhea. Normally, the average age at which women experience menopause is 52 years [1]. Early menopause can occur at ages under 40 due to various causes, such as chemotherapy, surgery, cancer, smoking, and infection, including mumps [2,3]. Increasing longevity among women and the increasing number of women diagnosed with early menopause make menopause an urgent health emergency [4]. If untreated, women can experience symptoms of menopause for decades. In menopause, ovarian production of estrogen is low; the low estrogen levels of menopause trigger troublesome systemic symptoms, including night sweats and osteoporosis, and local symptoms, e.g., vulvovaginal atrophy, dyspareunia, and other urogenital symptoms [1,3]. More serious risk factors include cardiovascular diseases, psychosexual dysfunction, neurological disorders, and premature death [2,5].

Hormonal replacement therapy (HRT) is the mainstay to control early and natural menopausal symptoms and reduce the risk factors [1,3]. HRT can be estrogen alone or a combination of estrogen and progestin [3]. Estradiol is an estrogen hormone used in HRT to alleviate early and natural menopausal symptoms [6]. Moreover, different pharmaceutical dosage forms have been available to deliver HRT, such as oral tablets, transdermal patches, vaginal creams, and intrauterine devices [1]. However, it has been reported that patients have non-compliance issues with oral estrogen preventive therapy for managing the systemic symptoms of menopause [4,7]. This is most likely brought on by oral estrogen therapy’s elevated risk of thrombosis and breast cancer [4].

Without estrogen replacement therapy, local symptoms, such as vaginitis, dryness, urinary discomfort, itchiness, irritability, or dyspareunia, can flare up [8]. Thus, vaginal estrogen therapy can help these women feel better and reduce their symptoms. Moreover, local estrogen delivery can improve sexual performance, vaginal surgery, and urine incontinence [7].

The vaginal route has been effectively utilized as an alternative to the oral route with many drugs, such as morphine, atropine, misoprostol, oxybutynin, and contraceptive hormones, to reduce systemic side effects and poor patient adherence [7,9].

The transdermal route has been employed for estradiol delivery, as it can bypass the first-pass metabolism. However, the horny skin layer is a formidable barrier against transdermal delivery, and the different regional skin thickness results in variable drug responses [7].

The vagina is a highly vascularized organ, and the vaginal route can bypass the first-pass metabolism and provides localized and rapid effects [7,9]. This is what has been termed the “first uterine pass effect”. When the drug is topically administered to the vagina, this provides first-hand/high local concentration in uterine tissues relative to low plasma recorded after vaginal delivery [10]. Vaginally administered estradiol has been reported to alleviate systemic menopausal symptoms, such as osteoporosis and hyperlipidemia linked to low estrogen levels. The risk of thrombosis and breast cancer from topical vaginal estrogen therapy is extremely low to nonexistent [4,11]. Recently, the topical administration of estrogen has been reported to alleviate the symptoms of postpartum dyspareunia and pelvic pain after normal baby delivery through the vagina [12].

On the downside, the residence duration of administered drugs in the vagina due to dilution by vaginal secretions and physiological movement of the vaginal wall is the main obstacle to successful vaginal drug delivery [9]. Additionally, the perception of patients is that the vaginally applied dose will “fall out” with gravity. These concerns may be valid, especially with the currently available options for topical vaginal estradiol, such as creams and vaginal tablets. Topical creams are associated with poor mucoadhesion and local irritation due to high concentrations of surfactants. Vaginal tablets, when disintegrated, may shed some particles and insoluble matter.

Adherence to the mucosal membrane is one crucial factor that significantly impacts how well vaginal delivery systems work. The mucoadhesive properties may enable prolonged residency and close contact with the administration site; thus, more time for drug absorption from vaginal mucosa can be allowed [9]. In general, it may be accepted that the stronger the mucoadhesion force of the polymer, the longer the drug formulation stays at the application site. Vaginal formulations’ ability to adhere to the mucosa is primarily influenced by the polymer’s physicochemical characteristics, such as molecular weight, level of ionization, and functional group [13]. However, preformed gels are not easy to adjust during administration as they depend on hand pressing to adjust the dose; therefore, in situ formed gels could be superior in this context. In situ gel has the convenience of being liquid, and the volume can be easily adjusted using a conventional vaginal applicator and quickly transformed into a gel. One of the commonly used in situ forming polymers and widely available is pluronic F127. At physiological temperature, the thermal gelation of pluronics typically occurs at high concentrations (typically >15% *w*/*w*). Gelation time is another factor that should be optimized. Any delay in the sol-to-gel transition may result in drug loss, since the gravity and secretions in the vagina quickly dilute any drug substance. We previously reported that hybrid chitosan-pluronic F127 could offer in situ gelation at physiological conditions with low concentrations of pluronic F127 and offer rapid gelation time [14].

Chitosan is a cationic polysaccharide obtained by the deacetylation of the natural polymer chitin. This cationic polymer is soluble and swellable in an acidic medium. These features make chitosan a suitable mucoadhesive polymer for vaginal delivery, as the normal vaginal pH is 3.8–4.2 [9,13].

Solid lipid nanoparticles (SLPs) have emerged as a more stable lipid-based nanosystem and alternative to phospholipid-based vesicle liposomes. SLPs can be fabricated from widely available and stable lipids, such as stearic acid, stearyl alcohol, cetostearyl alcohol, and glyceryl monooleate cetyl palmitate [15]. The inclusion of an insoluble drug such as estradiol into SLPs can offer enhanced solubility, increased surface area, and improved permeability for topical delivery [15]. SLPs have been useful for the vaginal delivery of poorly soluble drugs, such as ketoconazole, clotrimazole, and progesterone [16,17]. They have been successfully prepared and used for the vaginal delivery of antifungal drugs such as ketoconazole and clotrimazole. Polyoxyethylene (40) stearate has been used as a lipid-forming SLPS [16]. However, there are scarce reports on the utilization of SLPs for the vaginal delivery of estradiol to overcome the abovementioned vaginal barriers.

In this study, estradiol SLPs-based chitosan gels were prepared for vaginal delivery. SLPs were prepared from compritol ATO 888 and precirol. Moreover, chitosan was used as a mucoadhesive polymer to deliver estradiol into the vagina. Specific objectives included size, zeta potential, loading capacity, and morphological studies. The prepared SLPs-based gels were investigated for mucoadhesion, gelation time, and permeability through ex vivo mucosal membranes.

## 2. Results and Discussion

### 2.1. Compatibility Study

The FTIR spectra of estradiol, compritol 888 ATO, Precirol 5 ATO, and their physical mixtures were collected and are presented in Supplementary (Figure S1).

A strong characteristic band recorded at 3450 cm^−1^ was due to the stretching vibration of the phenolic (-OH) of estradiol. Bands from 3143 to 2800 cm^−1^ were due to CH_3_ and CH_2_ asymmetric and symmetric stretching vibrations. Medium bands from C=C stretching of the aromatic ring at 1616 to 1461 cm^−1^ were recorded. Data are presented in Supplementary Figure S1.

The two lipids, compritol 888 ATO (glyceryl dibehenate) and Precirol ATO 5 (glyceryl palmitostearate), showed major peaks at 1730 cm^−1^ due to –C = O stretching and between 2913 cm^−1^ and 2849 cm^−1^ due to –C–H stretching, and broad but weak peaks at 3450 to 3200 cm^−1^ due to free alcohol –OH of glyceryl moiety [18,19]. The characteristic peaks of the drug appeared but at lower intensities in the physical mixtures, and the FTIR spectra appeared as the superimposition of the individual spectra of the drug and lipids. These results indicated that no appreciable interactions could be detected between the drug and lipid-forming materials.

### 2.2. Preparation of Estradiol SLPs

**Figure 1:**
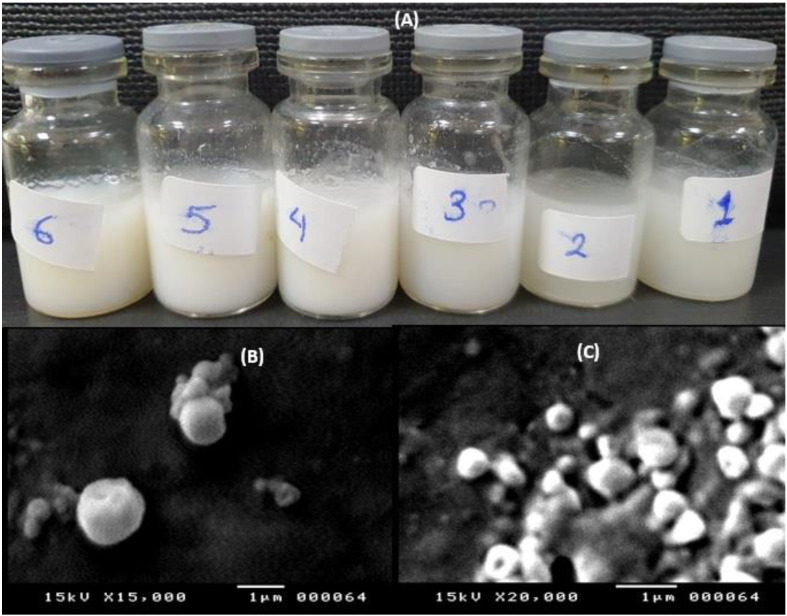
Macroscopic appearance of the prepared estradiol SLPS (**A**), SEM micrographs of F3 at different magnifications 15,000× (**B**) and 20,000× (**C**).

Six different formulations of SLP_S_ loaded with estradiol were composed of two different lipids, i.e., compritol 888 ATO and precirol ATO 5, and two stabilizers, namely pluronic F127 and tween 80. Pluronics are self-assembled triblock copolymers made of polyethylene oxide (PEO) and polypropylene (PPO) organized in a PEO-PPO-PEO structure. The hydrophobic and hydrophilic qualities are caused by the PPO and PEO parts, respectively. Pluronic F127’s stabilizing effect is assumed to be caused by the hydrophobic (PPO) component that adheres to the surface of the particles and the hydrophilic (PPO) component that spreads out into the aqueous media to offer steric shielding [20]. Tween 80 is one of the polysorbates with hydrophilic-lipophilic balance (HLB) = 15. It can stabilize the colloidal system and prevent SLPs from aggregation [21]. The emulsion/solvent evaporation process successfully produced F1–F6 with a macroscopic appearance of milky dispersion (Figure 1A).

### 2.3. Characterization of Estradiol-Loaded SLPs

The EE% was markedly high and ranged from 50% to 83%, as shown in Table 1. Different variables were studied, including lipid type (compritol and precirol) and stabilizers’ concentrations (pluronic = 100 and 200 mg and tween 80 = 0.5% and 1%). The highest EE% values were recorded for F1 (≈83%) and F3 (≈81%). In contrast, F4 and F6 showed the lowest EE% at 64 and 51%, respectively. There were no statistically significant (*p* > 0.05) differences in EE% for F1 and F3. Compritol and precirol are lipid-forming particles with the same melting point (60–73 °C) and HLB = 2. This could explain the comparable EE% recorded for compritol- (F1) and precirol (F3)-based SLPs. Further, increasing the amounts of pluronic F127 from 100 mg (F1 and F3) to 200 mg (F2 and F4) resulted in significant (*p* < 0.05) decreases in EE% (Table 1). Pluronic is a hydrophilic polymer that can stabilize nanoparticles through steric stabilization. However, larger amounts of pluronic saturate the nanoparticle surface and the excess amount can form micelles in the aqueous compartment, solubilize the poorly soluble drug estradiol, and decrease EE% for SLPs. Similarly, this could explain the decreases in EE% due to increased concentrations of the non-ionic surfactant tween 80 from 0.5% to 1%. Similar EE% (51–78%) was reported elsewhere with SLPs loaded with poorly soluble lipophilic drug alprazolam [22].

### 2.4. In Vitro Release

**Figure 2:**
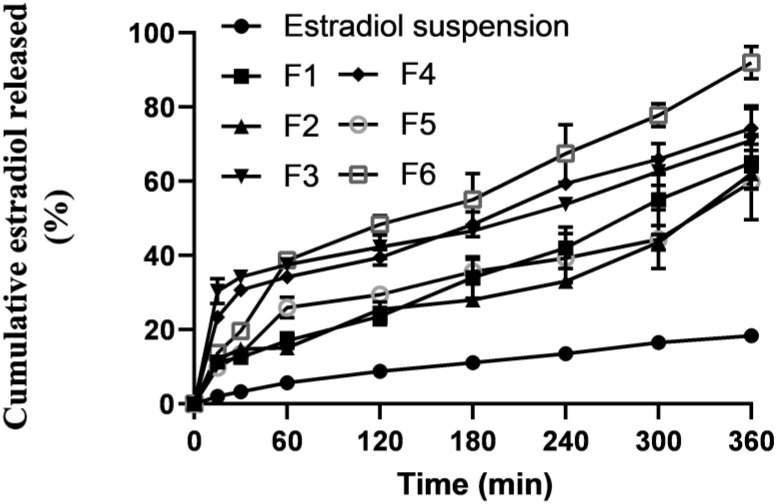
In vitro estradiol release profiles from the prepared estradiol SLPs and control estradiol suspension gels.

In vitro release profiles of estradiol from the prepared SLPs and control estradiol suspensions (Figure 2). Estradiol release was markedly enhanced from the prepared SLPs compared to those from estradiol solid suspension. The extremely slow release of estradiol from the control suspensions was as low as <20%, released over a 6 h-period. On the contrary, significant (*p* < 0.05) enhancement of estradiol release was exhibited by the prepared SLPs. Up to 4.4-fold increases in the release rates were recorded for SLPs compared to those from the solid suspensions. This significant enhancement of estradiol release from the prepared SLPs could be ascribed to being in a soluble form within SLPS and dispersed in an extremely large surface area due to the nanonization of the SLPs [23]. Additionally, 1.7-fold increases in the release rates were recorded for F6, containing a doubled concentration (1%) of tween 80, compared to F5, with the same lipid composition but a lower concentration (0.5%) of tween 80. Tween 80 is a highly hydrophilic and small molecular weight stabilizer that can easily partition out to the bulk of the aqueous phase and create channels within the lipospheres (SLPs), resulting in higher release rates. Except for F5, the general operating release kinetics best fit was zero-order kinetics (Table 2), showing that the drug release was independent of the remaining concentration in the SLPs. The estradiol release mechanism from F5 was the Higuchi model.

### 2.5. Characterization of Some Selected SLPs Gels

Two SLPs, F3 and F6, were selected for incorporation into two different concentrations (0.5 and 1%) of chitosan. The SLPs gels were characterized for gelation time, gelation temperature, mucoadhesion force, gelation strength, and viscosity at two different temperatures: 25 and 37 °C. Table 3 shows the results of characterization measurements for the prepared SLPs gels. The gelation temperature recorded for the four SLPs gels ranged from 31.5 to 34.2 °C. This range was considered below the physiological temperature of the vagina, 37.5 °C [24]. The gelation time measured for the SLPs was in the range of 23–34 s. This quick gelation transformation is likely to be desirable to minimize or prevent the leakage of the administered dose from the site of administration and minimize loss due to dilution by vaginal secretion. Another important parameter was the gelation strength. The values recorded for this parameter ranged from 26 to 32 s. Higher concentrations (1%) of chitosan generated stiffer and more cohesive gelation than the lower concentrations (0.5%). It has been reported that a gelation strength of more than 25 s helps the administered gel to keep its shape and resist rapid erosion of the gel. On the contrary, a gelation strength greater than 50 s might cause discomfort at the application site due to extensive stiffness [25]. The viscosity measurements, as shown in Table 3, were significantly dependent on the temperature and concentration of chitosan. Extensive gelation at 37 °C recorded viscosity values of around 21,000 mPa.s, compared to around 900 mPa.s. Similarly, the viscosity was concentration-dependent. Similar results have been reported elsewhere for hybrid gels of chitosan and pluronic F127 [14].

### 2.6. Ex Vivo Permeation Study

**Figure 3:**
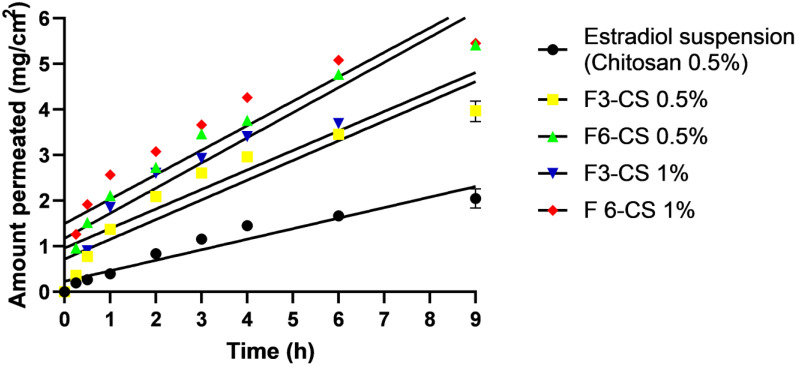
Ex vivo permeation profiles of estradiol from some selected SLPs gels and control estradiol suspension using excised ovine vaginal mucosal tissue; data points represent means ± D, n = 3.

Ex vivo permeation profiles of estradiol from SLPs hybrid gels and the control suspension gel are shown in Figure 3. A low and slow permeation profile was recorded for the control suspension, but higher and faster permeation profiles were recorded for all SLPs gels. The estimated permeation parameters quantitatively confirmed these results. For example, both flux and P_app_ recorded for the control estradiol suspension gel were 0.25 and 24.89 × 10^−2^ cm/min, while the recorded flux and P_app_ for the selected SLPs gels recorded 0.43−0.55 and 43−54.6 × 10^−2^ cm/min, respectively (Table 3). Incremental 1.7- to 2.2-fold increases in the permeation parameters for SLPs gels compared to the control suspension gel indicated significant (*p* < 0.05) permeation-enhancing effects of SLPs, which could be attributed to the solubilization of the SLPs to the lipophilic drug estradiol and hence enhancing permeation rates [26]. Furthermore, F6-CS 0.5% and F6-CS 1% showed superior permeability, compared to F3-CS 0.5% and F3-CS 1%. The only explanation that could be sought was due to the difference in the compositions of SLPs F3 and F6. Greater concentrations of tween 80 (1% *w*/*v*) were in favor of F6. Tween 80 is a hydrophilic surfactant with an HLB value of 15 that has been reported to enhance the permeability of poorly soluble drugs through biological membranes [27,28].

### 2.7. In Vitro MTT Assay for Vaginal Irritation Using Hela Cell Lines

**Figure 4:**
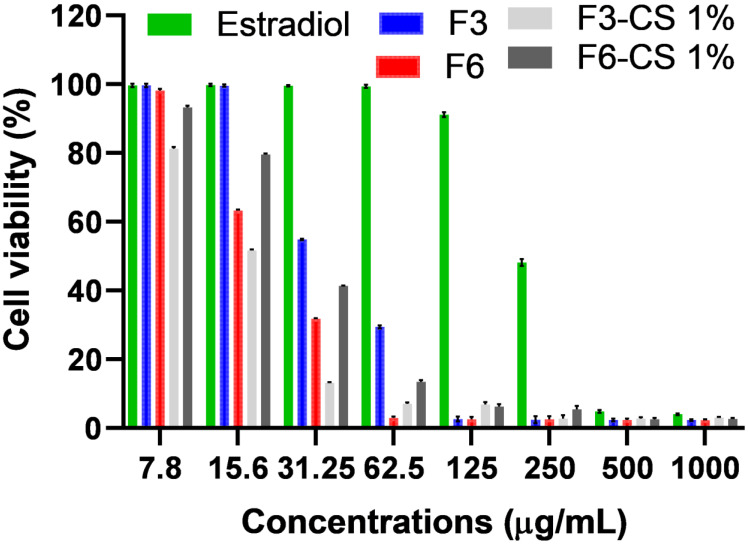
Cell viability (%) of Hela cell lines for different concentrations of the selected estradiol SLPs and estradiol suspension (control); data are represented as means ± SD, n = 5.

To investigate the irritation potential of the prepared SLPs loaded with estradiol on the vaginal mucosa, the selected SLPs, nanogels, and estradiol suspension (serving as a control) were studied using the MTT assay that was reported to be a potential in vitro irritation model to predict cell viability and correlated well with vaginal irritation [29]. The cell viability (%) recorded for the selected estradiol SLPs was significantly reduced at a concentration of ≤32.25 µg/mL for F3 and ≤15.62 µg/mL for F6 compared to that obtained from an estradiol suspension of ≤250 µg/mL (Figure 4). The higher cell viability (%) recorded for F3 compared to that obtained for F6 could be attributed to the composition of the prepared SLPs. F6 contained a doubled concentration (1% *w*/*v*) of the hydrophilic non-ionic surfactant tween 80, compared to that (0.5% *w*/*v*) contained in F3. Higher concentrations of tween 80 are likely to pose greater irritation potential. 

**Figure 5:**
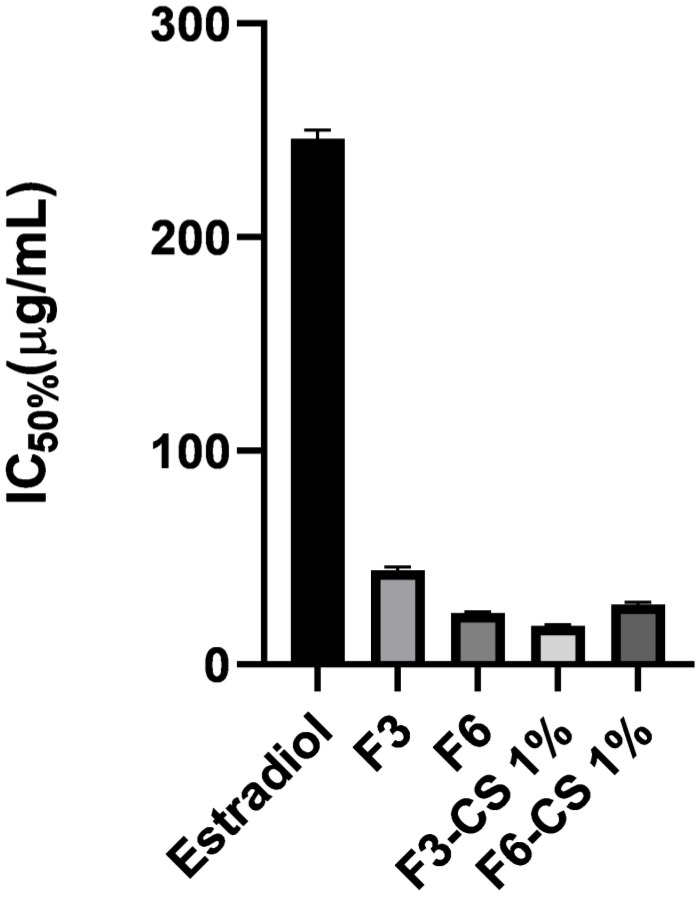
IC50% of the selected estradiol SLPs and estradiol suspension (control) using Hela cell lines; data are represented as means ± SD, n = 5.

More interestingly, chitosan-coated SLP F6-CS 1% recorded greater % cell viability than F6. These results indicated that the addition of chitosan reduced the irritation of SLN F6. The IC50% for F3, F6, F3-CS 1%, and F6-CS 1% recorded 43.6 µg/mL, 23.3 µg/mL, 18 µg/mL, 27.5 µg/mL and >31.25 µg/mL, respectively (Figure 5). On the contrary, coating the SLPs with the cationic polymer chitosan did not show a statistically (*p* > 0.05) significant reduction in irritation to F3 [30].

## 3. Materials and Methods

Estradiol was a gift provided by EIPICO Pharma Co., Cairo, Egypt. Compritol 888 ATO and precirol ATO 5 were a gift from Gattefosse Corporation, Lyon, France. Additionally, chitosan of high molecular weight (viscosity 800,000 cps; CAS 9012-76-4) and pluronic F127 were purchased from Sigma-Aldrich, St. Louis, MO, USA. Cell lines Hela-CCL-2 from tissue culture were purchased from VACSERA (Egyptian holding company for biological products and vaccines), Cairo, Egypt.

### 3.1. Compatibility Study

Spectral (FTIR) analyses were adopted to study the potential incompatibility between estradiol and lipids (solid lipid nanoparticle-forming materials) using a Thermoscientific Nicole IS 10, Waltham, MA, USA. Samples weighing 2–4 mg of estradiol, compritol 888 ATO, Precirol ATO 5 alone, and physical mixtures (PM) were compressed with potassium bromide in the form of a disc using a hydraulic press at 1 ton. The samples were scanned from 400 to 4000 cm^−1^. An FTIR spectrophotometer (Shimadzu IR-345, Kyoto, Japan) was employed to generate the spectra for the abovementioned samples.

### 3.2. Preparation of Estradiol SLPs

An altered emulsion/solvent evaporation [31] procedure was used to generate SLPs loaded with estradiol according to the composition shown in Table 4. The organic phase was prepared by the addition of 10 mg of estradiol and the lipid (compritol or precirol) to 5 mL of methanol, then the organic phase was heated to 75 °C. Tween 80 and pluronic F127 were dissolved in 10 mL of distilled water. The final solution was heated to 75 °C. The organic and aqueous phases were combined dropwise, and the emulsion was then mechanically agitated for five minutes to evaporate the methanol. The prepared estradiol-loaded SLNs were sonicated for 10 min and then homogenized for 5 min at 19,000 rpm. After that, the sonication process was repeated for 20 min.

### 3.3. Characterization of Estradiol-Loaded SLPs

#### 3.3.1. SLPs Size, PDI, and Zeta Potential Measurements

Malvern Zetasizer was used to determine the SLPs’ mean diameter, PDI, and zeta potential by employing the dynamic light scattering technique. The prepared estradiol-loaded SLPs were diluted by 1 × 10^−2^, and measurements were carried out three times.

#### 3.3.2. SEM

Selected SLPS were imaged using a Scanning Microscope JEOL-JSM6510 LA, JEOL, Tokyo, Japan. A gold sputter (SPI-Module Sputter Coater, SPI Supply Inc., West Chester, PA, USA) was employed to coat the samples on carbon stubs before being scanned under the microscope at a 5 kV acceleration voltage.

#### 3.3.3. Entrapment Efficiency (EE) (%)

The prepared estradiol-loaded SLPs were purified using a centrifuge (Hettich Zentrifugen, Tuttlingen, Germany) at 15,000× *g* for 90 min at 4 °C. The SLNs were then washed twice with distilled water, and the amount of free estradiol was determined by analyzing the supernatant with a UV spectrophotometer (Shimadzu, Kyoto, Japan) at 282 nm. Equation (1) was used to estimate the percentage of entrapped estradiol (%EE):

(1) $$\mathrm{EE}\%=\frac{Total\;amount\;of\;drug-amount\;of\;free\;unentrapped\;drug\;}{Total\;amount\;of\;drug\;}\times 100$$

The separated estradiol-loaded SLPs were placed in the donor compartment using a small glass cylinder reinforced on one side with a paddle-style USP dissolving equipment and encased from another with a dialysis membrane (molecular weight cut-off 12–14 KDa). The paddle was agitated at 100 rpm.

As the receptor was medium, 100 mL of SVF (pH 4.6) was employed. The medium’s temperature was carefully controlled at 37–0.50 °C, and 1% of tween 80 was added to maintain a sink condition. One ml samples were taken out and exchanged for an equal amount of fresh medium. Then, these samples were spectrophotometrically examined at 282 nm. Four kinetics models (zero order, first order, Higuchi model, and Koresymer-Peppas equation) were used to fit the cumulative release data from the examined SLPs.

### 3.4. Preparation of SLPs-Loaded Chitosan Gels

Mucoadhesive estradiol and SLPs-loaded gels were prepared using the cold method, with a few modifications. The required amount of chitosan (0.5–1% *w*/*w*) was dissolved in simulated vaginal fluid (SVF) with continuous stirring until fully dissolved. SLPs were added to the chitosan solution, followed by pluronic F127 (10% *w*/*v*). Additionally, the mixture was refrigerated overnight before being constantly stirred for one hour.

### 3.5. Characterization of SLPs Gels

#### 3.5.1. Viscosity Measurements

Using a rotational viscometer (Brookfield DV-II, Middleboro, MA, USA) fitted with a spindle LV-2, the viscosity of the produced gels was measured at two different temperatures: room temperature and 37 °C.

#### 3.5.2. Gelation Temperature

Using the visual inspection method, the gelation temperature of the generated gels was determined [14,25]. A volume (5 mL) of gel was added to a small beaker with a thermometer immersed. The temperature of the beaker was gradually increased at a rate of 1 °C/min while stirring at 50 rpm. The magnetic bar was not allowed to continue stirring, and the temperature of the gelation was noted.

#### 3.5.3. Gelation Time

Five ml of mucoadhesive gels were transferred into a small beaker and magnetically stirred at 34 °C and 50 rpm. After the movement of the magnetic bar ceased, the gelation time was calculated [14,25].

#### 3.5.4. Mucoadhesive Strength

The force required to detach the gel that acts as a mucoadhesive from the mucosa is known as mucoadhesive strength. A sheep’s intact vaginal mucosal membrane tissue was collected from a local slaughterhouse within an hour of the animal’s death. Following isolation, saline solution was used to wipe the vaginal mucosal membrane. Each around 2.5 cm^2^-sized vaginal mucosa fragment was affixed to a glass slide using cyanoacrylate adhesive and then secured to the bottom of the pan balance. The two vaginal mucosa fragments might have faced each other since a second glass slide was linked to the balance inverted. Five minutes were spent holding a weight (1 gm) of mucoadhesive gel between the tissues before progressively adding more weight into the other pan until the vaginal mucosa was disengaged. Equation (2) was used to work out the mucoadhesive force:

(2) $$\mathrm{Mucoadhesive strength}({\text{dyne/cm}}^{2})=\frac{W\;\times \;g\;\times 100\;}{S}$$

where S is the area (cm^2^) of the vaginal mucosa under examination, g is the acceleration due to gravity, and W is the weight necessary for detachment (in grams).

#### 3.5.5. Gel Strength

A 50-mL measuring cylinder was filled with 25 gm of the mucoadhesive geland and was gelled at 37 °C. The time required for a weight of 20 g to deeply penetrate 5.0 cm in the gel was then measured to establish the gel’s strength [25].

### 3.6. Ex Vivo Permeation Study

Fresh sheep vagina tissues were collected from a local slaughterhouse within 1 h of slaughter. The excised tissues of intact vaginal mucosa were isolated from the collected tissues and kept in normal saline till further use. In the same test conditions as the in vitro release study, the mucosa was used for the permeation study instead of the dialysis cellophane membrane.

Portions of the isolated vaginal mucosa with a surface area of 5 cm^2^ were used to envelop an open-sided tube; the mucosa was attached to the tube on one side with cyanoacrylate adhesive and strengthened on the other with a paddle-style USP dissolving equipment, which was agitated at 100 rpm. The donor compartment received 3 mL of estradiol suspensions, gels, or SLPs gel containing 10 mg of estradiol. The amount of estradiol permeated (mg/cm^2^) from samples of 3 mL at various intervals was spectrophotometrically analyzed. As the receptor medium, 100 mL of SVF (pH 4.6) was employed. The medium’s temperature was carefully maintained at 37 ± 1 °C, and 1% of tween 80 was added to establish sink conditions. At time intervals, as mentioned above, samples of 1 mL were withdrawn and replaced with the same volume of a new medium. The samples that were withheld underwent spectrophotometric evaluation at 282 nm. Estimates were made for the permeability parameters flow and apparent permeability coefficient (P_app_). The slope of the amount penetrated per cm^2^ vs. the time curve’s best-fitting line was called flux, and P_app_ was calculated using the equation shown below [32]:

(3) $$P_{\mathrm{app}}=\frac{F}{C}$$

where *F* is the flux, and *c* is the initial concentration (mg/mL).

### 3.7. In Vitro MTT Assay for Vaginal Irritation Using Hela Cell Lines

To create a full monolayer of a cellular sheet, 1 × 10^5^ cells/mL was added to a 96-well plate and incubated at 37 °C for 24 h. After the confluent sheet of cells had formed, the growth material was decanted from the 96-well plates, and the cell monolayer was washed twice by washing the media. The test sample was diluted twice in RPMI medium with 2% serum. Each dilution’s aliquot of 0.1 mL was examined in a different well. The 96-well plates were incubated at 37 °C. Additionally, the MTT solution was prepared (5 mg/mL). To each well, 20 uL of the MTT solution was added. For five minutes, the medium was shaken at 150 rpm on a shaking table to completely incorporate the MTT. The MTT was allowed to be digested for 4 h in an incubator (37 °C, 5% CO_2_) and then discarded with the media. Formazan, an MTT metabolic product, was redissolved in 200 ul DMSO and was shaken for 5 min at 150 rpm to properly combine the formazan and solvent. At 620 nm, the background was subtracted from the optical density measurement at 560 nm.

### 3.8. Statistical Analysis

GraphPad Prism 8.4.3 software was utilized to study the statistical analysis of the results. Moreover, Student’s *t*-test was used to compare two groups, and a two-way ANOVA was used to compare three or more groups.

## 4. Conclusions

Local symptoms of menopause can be in the form of vaginitis, dryness, urinary discomfort, itchiness, irritability, or dyspareunia. Topical vaginal estrogen therapy could be superior to oral hormonal replacement therapy. Topical vaginal delivery of estradiol has been linked to low to no risk of thrombosis and breast cancer. Additionally, vaginal creams are messy, have poor mucoadhesion, and can be diluted and flushed with vaginal secretions. This study investigated and characterized chitosan-coated SLPs loaded with estradiol. The SLNs recorded nanosizes of 420–850 nm and faster release rates by up to 4.4 folds compared to that from the control suspension. The prepared nanogels demonstrated superior mucoadhesion and permeability through the vaginal mucosa. The optimized hybrid SLN/chitosan gels recorded thermal gelation, superior mucoadhesion, and up to 2.2-fold increases in the permeation of the poorly soluble drug through excised vaginal mucosa tissues compared to the control with acceptable tolerability, as was evident from the in vitro irritation model (the MTT assay). These key findings warrant the prepared hybrid SLP/chitosan gels as potential drug delivery systems for the vaginal delivery of estradiol.

## Figures and Tables

**Table 1:** Characterization features of the prepared SLPs loaded with estradiol; results are expressed as mean ± SD.

Formula No.	% EE	% Cumulative Release	Size (nm)	PDI	Potential (mV)
F1	82.72 ± 2.0	68.90 ± 4.3	849.9 ± 5.5	0.64 ± 0.2	−10.9 ± 1.4
F2	70.67 ± 2.5	59.06 ± 3.4	713.5 ± 5.7	0.55 ± 0.25	−9.9 ± 1.0
F3	80.58 ± 3.0	77.05 ± 4.4	504 ± 3.0	0.59 ± 0.15	−5.7 ± 2.2
F4	63.60 ± 2.0	78.56 ± 5.4	423.3 ± 4.5	0.52 ± 0.12	−4.6 ± 1.3
F5	72.87 ± 2.4	52.56 ± 4.5	680.5 ± 4.3	0.57 ± 0.08	−12.0 ± 1.4
F6	50.82 ± 3.5	88.92 ± 5.5	450.7 ± 3.5	0.54 ± 0.1	−15.1 ± 1.2

**Table 2:** Release rate constants (K), regression coefficient (R2), and release exponent estimated using four kinetic release models for estradiol SLPs.

Formulation	0th Order		1st Order		Higuchi		Korsmeyer-Peppas		
	R^2^	K (%.min^−1^)	R^2^	K (min^−1^)	R^2^	K (%.min^−0.5^)	R^2^	K (%.min^−n^)	*n* *
F1	0.984	0.14	0.96	0.01	0.94	2.6	0.6	0.42	1
F2	0.955	0.09	0.952	0.009	0.929	2.1	0.56	0.7	0.99
F3	0.94	0.18	0.899	0.01	0.887	2.1	0.55	2.7	0.98
F4	0.989	0.148	0.978	0.013	0.967	3.1	0.669	1.7	1.5
F5	0.960	0.14	0.981	0.01	0.986	3.03	0.72	1.7	1.4
F6	0.95	0.23	0.990	0.022	0.992	4.91	0.74	2.5	1.5

* *n* denotes the release exponent.

**Table 3:** Gelation time, gelation temperature, mucoadhesion force, viscosity measurements, and permeation parameters for the prepared SLPs-based hybrid gels; data are represented as means ± SD, n = 3.

SLPs Hybrid Gels	Gelation Time (s)	Gelation Temp	Mucoadhesion Force (g/cm^2^)	Gel Strength (S)	Viscosity (mPa.S)		Permeation Parameters	
					25 °C	37 °C	Flux	P_app_ × 10^−2^ Cm/min)
**F3-CS 0.5%**	23 ± 2.0	33.7 ± 1.6	31.72 ±	26 ± 2.0	540 ± 20	16,165 ± 100	0.43 ± 0.02	43 ± 2.0
**F6-CS 0.5%**	25 ± 3.0	34.2 ± 1.4	32.21 ±	28 ± 2.0	517 ± 18	14,812 ± 150	0.55 ± 0.03	54.6 ± 3.5
**F3-CS 1%**	30 ± 4.0	31.5 ± 1.2	36.92 ±	32 ± 4.0	872 ± 35	20,865 ± 200	0.44 ± 0.01	43.86 ± 2.4
**F6-CS 1%**	34 ± 5.0	31.9 ± 2.1	36.13 ±	31 ± 3.0	923 ± 40	21,322 ± 250	0.53 ± 0.02	52.78 ± 4.3

**Table 4:** Composition of the prepared SLPs.

Formulation No.	Composition (mg)			
	Compritol 888 ATO (mg)	Precirol ATO 5 (mg)	Pluronic F127 (mg)	Tween 80 (%)
F1	200	-	100	0.5
F2	200	-	200	0.5
F3	-	200	100	0.5
F4	-	200	200	0.5
F5	200	200	200	0.5
F6	200	200	100	1

## Data Availability

Data is contained within the article and supplementary material.

## Supplementary Materials

The following supporting information can be downloaded at: https://www.mdpi.com/article/10.3390/ph16091284/s1, Figure S1: FTIR spectra of estradiol (**A**), Compritol 888 ATO (**B**), Precirol ATO 5 (**C**), estradiol:Compritol 888ATO PM (**D**) and estradiol:Precirol ATO 5 (**E**).

## References

[1] Paciuc J., Deligdisch-Schor L., Miceli A.M. (2020). Hormone therapy in menopause. Advances in Experimental Medicine and Biology.

[2] Huan L., Deng X., He M., Chen S., Niu W. (2021). Meta-analysis: Early Age at Natural Menopause and Risk for All-Cause and Cardiovascular Mortality. BioMed Res. Int..

[3] Faubion S.S., Kuhle C.L., Shuster L.T., Rocca W.A. (2015). Long-term health consequences of premature or early menopause and considerations for management. Climacteric.

[4] Mikkola T.S., Tuomikoski P., Lyytinen H., Korhonen P., Hoti F., Vattulainen P., Gissler M., Ylikorkala O. (2016). Vaginal estradiol use and the risk for cardiovascular mortalit. Hum. Reprod..

[5] Okeke T.C., Anyaehie U.B., Ezenyeaku C.C. (2013). Premature menopause. Ann. Med. Health Sci. Res..

[6] Luine V. (2014). Estradiol and cognitive function: Past, present and future. Horm Behav..

[7] Krause M., Wheeler T.L., Snyder T.E., Richter H.E. (2009). Local Effects of Vaginally Administered Estrogen Therapy: A Review. J. Pelvic Med. Surg..

[8] Cardozo L., Bachmann G., McClish D., Fonda D., Birgerson L. (1998). Meta-analysis of estrogen therapy in the management of urogenital atrophy in postmenopausal women: Second report of the Hormones and Urogenital Therapy Committee. Obstet. Gynecol..

[9] Osmałek T., Froelich A., Jadach B., Tatarek A., Gadziński P., Falana A., Gralińska K., Ekert M., Puri V., Wrotyńska-Barczyńska J. (2021). Recent Advances in Polymer-Based Vaginal Drug Delivery Systems. Pharmaceutics.

[10] Bulletti C., de Ziegler D., Flamigni C., Giacomucci E., Polli V., Bolelli G., Franceschetti F. (1997). Targeted drug delivery in gynaecology: The first uterine pass effect. Hum. Reprod..

[11] Archer D.F., Kimble T.D., Lin F.Y., Battucci S., Sniukiene V., Liu J.H. (2018). A Randomized, Multicenter, Double-Blind, Study to Evaluate the Safety and Efficacy of Estradiol Vaginal Cream 0.003% in Postmenopausal Women with Vaginal Dryness as the Most Bothersome Symptom. J. Women’s Health.

[12] Smith P.E., McLaughlin E.M., Pandya L.K., Hade E.M., Lynch C.D., Hudson C.O. (2022). A pilot randomized controlled trial of vaginal estrogen on postpartum atrophy, perineal pain, and sexual function. Int. Urogynecology J..

[13] Roy S., Pal K., Anis A., Pramanik K., Prabhakar B. (2009). Polymers in Mucoadhesive Drug-Delivery Systems: A Brief Note. Des. Monomers Polym..

[14] Fathalla Z., Mustafa W.W., Abdelkader H., Moharram H., Sabry A.M., Alany R.G. (2022). Hybrid thermosensitive-mucoadhesive *in situ* forming gels for enhanced corneal wound healing effect of L-carnosine. Drug Deliv..

[15] Liu M., Wen J., Sharma M. (2020). Solid Lipid Nanoparticles for Topical Drug Delivery: Mechanisms, Dosage Form Perspectives, and Translational Status. Curr. Pharm. Des..

[16] Cassano R., Ferrarelli T., Mauro M.V., Cavalcanti P., Picci N., Trombino S. (2014). Preparation, characterization and in vitro activities evaluation of solid lipid nanoparticles based on PEG-40 stearate for antifungal drugs vaginal delivery. Drug Deliv..

[17] Cassano R., Trombino S. (2019). Solid Lipid Nanoparticles Based on L-Cysteine for Progesterone Intravaginal Delivery. Int. J. Polym. Sci..

[18] Ali A., Madni A., Shah H., Jamshaid T., Jan N., Khan S., Khan M.M., Mahmood M.A. (2023). Solid lipid-based nanoparticulate system for sustained release and enhanced in vitro cytotoxic effect of 5-fluorouracil on skin Melanoma and squamous cell carcinoma. PLoS ONE.

[19] Singh H., Jindal S., Singh M., Sharma G., Kaur I.P. (2015). Nano-formulation of rifampicin with enhanced bioavailability: Development, characterization and in vivo safety. Int. J. Pharm..

[20] Chong J.Y., Mulet X., Waddington L.J., Boyd B.J., Drummond C.J. (2011). Steric stabilisation of self-assembled cubic lyotropic liquid crystalline nanoparticles: High throughput evaluation of triblock polyethylene oxide-polypropylene oxide- polyethylene oxide copolymers. Soft Matter.

[21] Göppert T.M., Müller R.H. (2005). Polysorbate-stabilized solid lipid nanoparticles as colloidal carriers for intravenous targeting of drugs to the brain: Comparison of plasma protein adsorption patterns. J. Drug Target..

[22] Rao H., Ahmad S., Madni A., Rao I., Ghazwani M., Hani U., Umair M., Ahmad I., Rai N., Ahmed M. (2022). Compritol-Based Alprazolam Solid Lipid Nanoparticles for Sustained Release of Alprazolam: Preparation by Hot Melt Encapsulation. Molecules.

[23] Mura P., Maestrelli F., D’ambrosio M., Luceri C., Cirri M. (2021). Evaluation and Comparison of Solid Lipid Nanoparticles (SLNs) and Nanostructured Lipid Carriers (NLCs) as Vectors to Develop Hydrochlorothiazide Effective and Safe Pediatric Oral Liquid Formulations. Pharmaceutics.

[24] Abrams R.M., Royston J.P. (1981). Some Properties of Rectum and Vagina as Sites for Basal Body Temperature Measurement. Fertil. Steril..

[25] Mura P., Mennini N., Nativi C., Richichi B. (2018). In situ mucoadhesive-thermosensitive liposomal gel as a novel vehicle for nasal extended delivery of opiorphin. Eur. J. Pharm. Biopharm..

[26] Ghasemiyeh P., Mohammadi-Samani S. (2020). Potential of Nanoparticles as Permeation Enhancers and Targeted Delivery Options for Skin: Advantages and Disadvantages. Drug Des. Dev. Ther..

[27] Yasir M., Som I., Bhatia K. (2012). Status of surfactants as penetration enhancers in transdermal drug delivery. J. Pharm. Bioallied Sci..

[28] Akhtar N., Rehman M.U., Khan H.M.S., Rasool F., Saeed T., Murtaz G. (2011). Penetration Enhancing Effect of Polysorbate 20 and 80 on the In Vitro Percutaneous Absorption of LAscorbic Acid. Trop. J. Pharm. Res..

[29] Ayehunie S., Cannon C., LaRosa K., Pudney J., Anderson D.J., Klausner M. (2011). Development of an in vitro alternative assay method for vaginal irritation. Toxicology.

[30] Frigaard J., Jensen J.L., Galtung H.K., Hiorth M. (2022). The Potential of Chitosan in Nanomedicine: An Overview of the Cytotoxicity of Chitosan Based Nanoparticles. Front. Pharmacol..

[31] Nabi-Meibodi M., Vatanara A., Najafabadi A.R., Rouini M.R., Ramezani V., Gilani K., Etemadzadeh S.M.H., Azadmanesh K. (2013). The effective encapsulation of a hydrophobic lipid-insoluble drug in solid lipid nanoparticles using a modified double emulsion solvent evaporation method. Colloids Surf. B Biointerfaces.

[32] Mohamad S.A., Mustafa W.W., Salem H., Elrehany M., Rofaeil R.R., Abdelkader H. (2022). Physicochemical characteristics and ex vivo skin permeability for three phosphodiesterase 5 inhibitors (sildenafil, tadalafil and vardenafil): A proof-of-concept study for topical penile therapy. J. Drug Deliv. Sci. Technol..

