# Supplementary material for: Chitosan/Solid-Lipid Nanoparticles Hybrid Gels for Vaginal Delivery of Estradiol for Management of Vaginal Menopausal Symptoms
Source: Pharmaceuticals (Basel). 2023 Sep 11;16(9):1284. doi: 10.3390/ph16091284 (PMC10536129; doi:10.3390/ph16091284)
Supplement: Supplementary file 1 [file pharmaceuticals-16-01284-s001.zip › pharmaceuticals-2583358-supplementary.pdf]

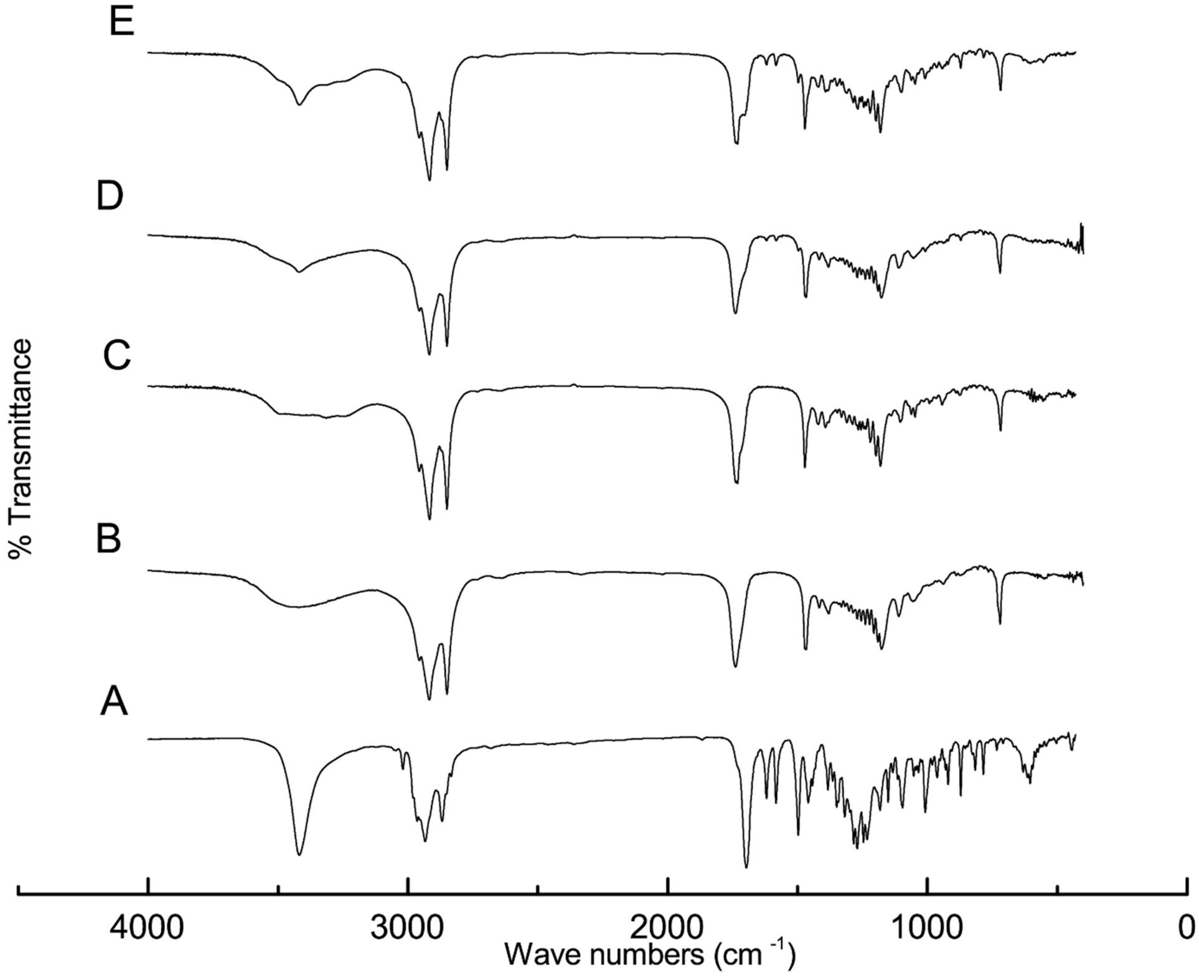

Figure S1: FTIR spectra of estradiol (**A**), Compritol 888 ATO (**B**), Precirol ATO 5 (**C**), estradiol:Compritol 888ATO PM (**D**) and estradiol:Precirol ATO 5 (**E**).
